# Where Do the Eyes Really Go in the Hollow-Face Illusion?

**DOI:** 10.1371/journal.pone.0044706

**Published:** 2012-09-04

**Authors:** Marc Grosjean, Gerhard Rinkenauer, Stephanie Jainta

**Affiliations:** Leibniz Research Centre for Working Environment and Human Factors, Dortmund, Germany; University of Melbourne, Australia

## Abstract

The hollow-face illusion refers to the finding that people typically perceive a concave (hollow) mask as being convex, despite the presence of binocular disparity cues that indicate the contrary. Unlike other illusions of depth, recent research has suggested that the eyes tend to converge at perceived, rather than actual, depths. However, technical and methodological limitations prevented one from knowing whether disparity cues may still have influenced vergence. In the current study, we presented participants with virtual normal or hollow masks and asked them to fixate the tip of the face’s nose until they had indicated whether they perceived it as pointing towards or away from them. The results showed that the direction of vergence was indeed determined by perceived depth, although vergence responses were both somewhat delayed and of smaller amplitude (by a factor of about 0.5) for concave than convex masks. These findings demonstrate how perceived depth can override disparity cues when it comes to vergence, albeit not entirely.

## Introduction

Viewing the inside of a mask of a (concave) face typically results in the perception of a forward facing (convex) face. This hollow-face illusion [1], as it is known, persists despite people’s explicit knowledge about the shape of the mask and the presence of unambiguous binocular disparity information that indicates that the surface of the mask is actually concave. Although there are many, sometimes ambiguous, monocular and binocular depth cues present in a hollow mask, it is the arrangement of monocular pictorial cues that indicate the presence of a face that is thought to induce the illusion of depth [1]–[3]. According to classical accounts, this only occurs when reactivated knowledge about faces dominates over binocular disparity information [1]. More recent views have also tried to account for the illusion by noting that people tend to show a general convexity preference when faced with ambiguous incoming depth information [4].

Beyond trying to understand why this perceptual illusion occurs, recent research has also sought to determine whether and how this illusion influences action control as well. Relying on the binocular presentation of virtual normal and hollow faces, Hartung, Schrater, Bülthoff, Kersten, and Franz [5] asked participants to point and touch the nose or cheek of the face, or to verbally estimate their distance. Consistent with the perception of a forward facing face, participants always estimated noses as being closer to them than cheeks and this by similar amounts in the verbal and manual tasks. This indicates that both perception and action were subject to the illusion. However, the estimated distances between nose and cheek were more similar for hollow than normal faces, which suggests that the integration of binocular disparity information was probably attenuating the illusion.

Króliczak, Heard, Goodale, and Gregory [6] investigated the same question, but also included a critical condition in which participants were asked to rapidly “flick” away a small object that had been placed on the cheek or forehead of a mould of a normal or hollow face. In contrast to Hartung et al. [5], participants performed accurate flicking movements for both normal and hollow faces, despite experiencing the illusion. The fact that such actions are immune to the illusion was taken as evidence for the operation of two visual processing streams, one for conscious perception, which is subject to such illusions, and one for rapid object-oriented actions, which is not [7]. Króliczak et al. attributed the effects obtained by Hartung et al. to their use of relatively slow pointing movements that would have provided enough time for (illusory) conscious perception to influence their manual actions.

Of particular interest here, Króliczak et al. [6] also proposed that one veridical cue participants may have relied upon to guide their flicking movements is vergence. Indeed, vergence has been shown to be used in reaching [8] and binocular disparity is often thought to be the primary input to stimulate vergence responses [9], [10]. Hoffmann and Sebald [11] tried to address this issue by tracking where the eyes go in the hollow-face illusion. They asked participants to fixate the nose of a real normal or hollow mask and found that mean vergence responses were directed to similar positions in space whether people experienced the illusion or were looking at a normal face. In other words, vergence eye movements were being driven by perceived depth, independent of binocular disparity cues. This suggests that vergence may not have been the cue relied upon by participants to guide their accurate flicking movements in Króliczak et al.’s study.

Hoffmann and Sebald’s [11] findings are consistent with others that have shown that disparity is not necessary to induce vergence responses, as shown for both monocular [12] and binocular [13], [14] viewing conditions. However, because perceived depth was correlated with depth cues in most of those studies, it is unclear whether the vergence movements were induced by perceived depth itself [15]–[17]. What most studies seem to reveal is that disparity cues typically drive vergence responses when monocular and binocular depth cues are in conflict. Furthermore, when vergence responses are found to be induced by perceived depth and/or monocular depth cues, they are generally weaker and of smaller amplitude than under viewing conditions including binocular disparity. However, it should be noted that none of these studies employed a depth illusion induced by monocular pictorial cues, such as the ones present in hollow masks.

Although Hoffmann and Sebald’s [11] experiment provides a valuable first step in characterizing vergence eye movements during the hollow-face illusion, it also has a number of limitations. Their eye tracker only allowed them to record rather coarse changes in vergence and they only had one trial per participant and condition. Moreover, in the absence of any clearly defined time of mask onset they could only analyze late vergence eye movements, which they did by taking an average over a relatively long time interval (5 s). Thus, it is very difficult to determine whether vergence was actually affected by binocular disparity or not. As they acknowledge themselves, it is possible that the eyes initially diverged in the direction of the disparity-defined depth of the actual concave face and then, as reactivated face knowledge started to drive perception, converged towards the perceived depth of the face. Such initial vergence responses would be consistent with Króliczak et al.’s [6] proposal.

The aim of the present study was to examine the precise time course of such vergence responses and, in particular, to determine whether the eyes initially diverge when people experience the hollow-face illusion. Based on Hoffmann and Sebald’s [11] results, one would expect vergence responses to ultimately converge at the same depth for normal and hollow masks. However, it remains open whether the presence of binocular disparity information actually influences early vergence responses in such situations, which is necessary to fully understand the influence perception has on vergence eye movements.

To investigate this issue, we presented participants with virtual (hollow) masks and asked them to fixate the tip of the nose until they had reported whether they perceived it as pointing towards or away from them. We employed virtual, instead of real, masks to increase the level of experimental control (e.g., regarding timing and illumination). In addition to employing a high-resolution eye tracker, we also included a vergence step-stimulus task which involved presenting stimuli at different depths relative to the display plane by only changing their absolute disparity, while keeping accommodation constant [18], [19]. The rationale was that if observers could perform this task accurately, vergence responses during the illusion could not be attributed to an inability to respond to convergent and divergent disparity cues with an appropriate vergence adjustment.

## Methods

### Ethics Statement

The study was conducted according to the code of ethics of the World Medical Association (Declaration of Helsinki) and was approved by the ethics committee of the Leibniz Research Centre of Working Environment and Human Factors. All participants gave written informed consent prior to participation and all data were analyzed anonymously.

### Participants

Twenty-three young adults (mean age = 23.9 years; age range = 20–28 years) took part in the study in exchange of monetary compensation. No participant wore glasses during the experiment. Visual acuities were better than 1 (decimal units) in each eye and stereo-thresholds were better than 60 arcsec (TNO Test). Moreover, all participants successfully passed an additional pre-test in which they had to identify crossed and uncrossed stereo disparities of 15 arcmin that were presented with the same stereoscopic technique that was employed in the actual experiment (the stimuli were similar to those used in the short vergence test described below).

### Apparatus and Stimuli

Viewing distance was held constant at 60 cm, which corresponds to an initial vergence demand of 6°. This was achieved by using a chin-and-head rest that also contained temporal rests to further minimize head movements and potential influences from the vestibular system [20]. Dichoptic presentation of visual stimuli was performed with 3D Vision wireless shutter glasses (NVIDIA Corp., Santa Clara, CA, USA) that were synchronized at 120 Hz (60 Hz for each eye) with a 22-inch color monitor (Samsung SyncMaster 2233RZ, Samsung Electronics, Korea). The glasses were attached to the chin-and-head rest and were positioned about 3 cm in front of the participants’ eyes. Eye movements were recorded with the video-based EyeLink II (sampling rate: 500 Hz), which tracks both eyes simultaneously with a theoretical noise-limited resolution of 0.6 arcmin and velocity noise of <3°/s for two-dimensional eye tracking (details provided by SR Research Ltd., Osgoode, ON, Canada). The EyeLink II cameras were also attached to the chin- and-head rest and were fixed just below the shutter glasses. Participants’ manual responses were recorded with a custom-made button box that contained two switches, one labeled “front” and the other “back” (see next section for details regarding the tasks).

**Figure 1:**
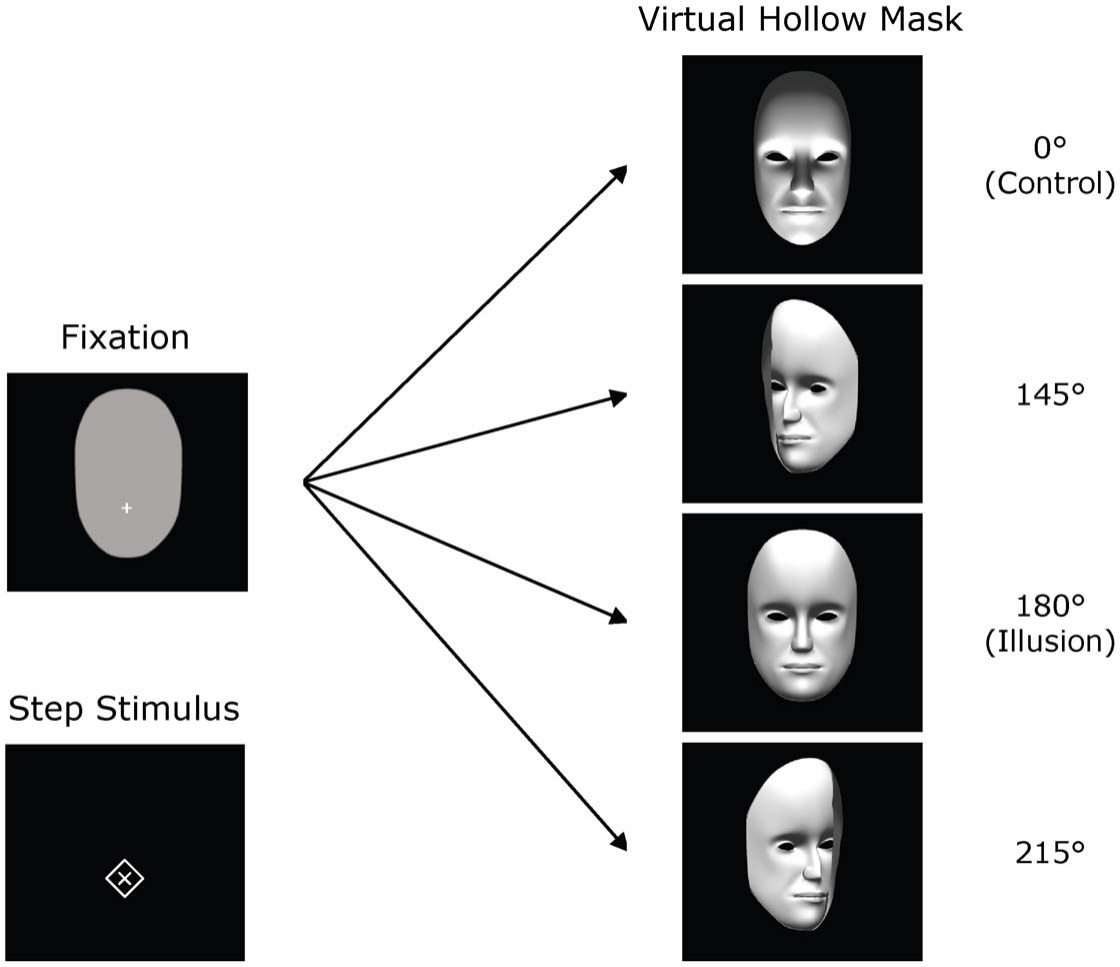
The fixation and virtual hollow-mask stimuli (presented without disparity information for illustration purposes). The 0° and 180° mask orientations correspond to the control and illusion conditions, respectively. The vergence step stimulus is also presented (see text for details).

Figure 1 presents the different stimuli that were used. The virtual hollow mask consisted of a 3D model that was rendered using the Vizard VR Toolkit (WorldViz LLC, USA). It was presented in 1 of 4 orientations: 0° (convex mask with the nose pointing towards the observer), 145° (concave mask with the nose pointing away from and slightly to the left of the observer), 180° (concave mask with the nose pointing away from the observer), and 215° (concave mask with the nose pointing away from and slightly to the right of the observer). The 0° and 180° orientations correspond to the control and illusion conditions, respectively. The remaining orientations (145° and 215°) were used as filler conditions to also include stimuli for which the likelihood of experiencing the illusion was reduced (see [11]).

Ambient lighting was set to 46 lux and the mask was presented in white (mean luminance = 94.5 cd/m^2^) on a dark background (0.6 cd/m^2^), with an invisible virtual light source positioned in front of and below the mask. The mask was rotated around a vertical axis that was placed at the center of the virtual head and at the horopter, which was set at the depth of the monitor. The distance between the axis of rotation (i.e., the horoptor) and the tip of the nose was 5.3 cm. Thus, the position of the tip of the nose was about 33 arcmin in front of the horopter for the control condition and about 28 arcmin behind the horopter for the illusion condition. Participants’ eye height was adjusted to be level with the tip of the mask’s nose. The mask display was preceded by a constant fixation display that contained a gray oval and a white (190 cd/m^2^) fixation cross (height×width: 1.0°×1.0°; line width: 6.5 arcmin) that were presented at the horopter (see Figure 1). To minimize changes in pupil size, the size and luminance of the oval were adjusted to approximate the average size (21.7°×13.8°) and luminance (94.5 cd/m^2^) of the hollow-mask stimuli. The fixation cross was placed centrally and at the same height as the tip of the mask’s nose (i.e., at participants’ eye height).

Figure 1 also contains the step stimulus that was used in a short vergence test administered at the end of the experiment. It consisted of a cross (1.9°×1.9°) surrounded by a diamond (2.9°×2.9°; both line widths: 6.5 arcmin) presented centrally and at participants’ eye height. The stimulus was presented in dark gray (38 cd/m^2^) on a dark background (same as above), and could appear at the horopter and 30 arcmin in front of or behind it.

The stimuli used to calibrate the eye tracker were presented at the horopter and at random locations within a 3×3 calibration grid. The grid was centered at the same location as the fixation cross (see above) and the horizontal and vertical distances between the calibration points were 4.8° and 3.7°, respectively. Stimuli consisted of filled squares that initially subtended 1.0°×1.0° and then shrank within 500 ms to a remaining cross of 0.3°×0.3° (line width: 1.6 arcmin). To approximate the same pupil sizes as in experimental trials, the stimuli were presented in white on the same gray oval that was used in the fixation display (see above).

### Design and Procedure

Participants performed 16 experimental blocks of 8 trials each, corresponding to 2 repetitions of each of the 4 mask orientations presented in a pseudorandom order. Thus, there was a total of 32 observations for each participant and condition. A trial started with the presentation of the fixation display for a randomly-chosen duration of 1001–1500 ms to avoid anticipatory eye movements (e.g., [21]). The fixation display was directly followed by the virtual hollow-mask display. Participants’ task was to fixate the tip of the mask’s nose and to report, at their leisure, whether they perceived the nose as pointing towards or away from them by pressing the “front” or “back” button, respectively. The mask remained on the screen for another 500 ms after the response and was then immediately followed by the fixation display for the next trial.

At the end of the experiment, participants were also administered a short vergence test. This involved 4 blocks of 8 trials each, which corresponded to 4 repetitions for each step-stimulus type (convergent, divergent) presented in a pseudorandom order. This resulted in 16 observations per participant and condition. Each trial started with the presentation of the step stimulus at the horopter for a random duration of 1001–1500 ms. The step stimulus then moved in front of or behind the horopter. The task of the participants was to continue to fixate the stimulus and indicate, at their leisure, whether it had moved towards or away from them by using the same button box as above. The step stimulus then remained where it was for another 500 ms before moving back to the horopter for the beginning of the next trial.

Before each block of the experiment and vergence test, the eye tracker was calibrated by binocularly presenting stimuli at random locations within the calibration grid. Participants were instructed to carefully fixate the stimulus and then to press either of the buttons to move on to the next stimulus. The stimulus remained visible for another 100 ms and was then replaced with the next calibration stimulus until all 9 locations had been used.

### Data Analysis

The main goal of this experiment was to compare vergence responses in the control and illusion conditions for those participants who actually experienced the illusion. Prescreening of the data revealed that 12 out of the 23 participants provided more than 50% front responses in both the control and illusion conditions. Defined in this way, the proportion of participants who experienced the illusion (∼50%) is comparable to that observed in related studies using real hollow masks (e.g., [11]). This definition also has the benefit of assuring that there will be enough illusion trials for the subsequent vergence analysis. The quality of the calibration data was too low to provide a sufficient number of valid measurements for 3 of the “illusion” participants. This was probably due to a lack of compliance on their part to fixate each of the calibration stimuli properly. Indeed, eye-tracking is always subject to some uncertainty that can be described by a standard deviation due to the calibration (SDc) [22].Generally, each calibration run included a routine to address this uncertainty (for further examples, see [23], [24]) and for the present data we excluded blocks of trials which showed a SDc larger than 10 arcmin. Thus, the sample size was further reduced to 9 and all subsequent analyses were performed on this subsample only.

For the behavioral (manual) data, the proportion of front button responses and mean response times (i.e., time from mask onset to button-press onset) were computed for each participant and condition. The former values were arcsine transformed to address the non-normality of proportions [25]. Both measures were then submitted to separate one-way repeated-measures analysis of variance (ANOVA) with mask orientation as a within-participants factor. When necessary, violations of the sphericity assumption were corrected for using the Greenhouse-Geisser ε.

For the vergence (ocular) data, the analyzes were restricted to front (manual) responses in the control and illusion conditions for experimental blocks, and to correct (manual) responses (almost all trials) for the vergence-test blocks. Eye-movement trajectories were segmented into trial epochs going from 50 ms before mask (step-stimulus) onset to 500 ms after the button press. Baseline was defined as the 50-ms interval from trial epoch onset to mask (step-stimulus) onset. Saccades were detected using a velocity-based algorithm [26] that was applied to the conjugate eye movement signals ([Left eye + Right eye]/2) and adjusted to detect saccades with amplitudes larger than about 0.3° (for more details, see [27]). All trial epochs containing blinks or saccades within the first critical 550 ms were then eliminated. After applying these selection criteria, the number of valid trials was reduced by about 55%. It is worth noting here that saccades are often observed in tasks that were designed to only induce vergence eye movements [28]–[31]. Moreover, given that the (mask) stimulus for vergence was relatively weak and participants were not strictly instructed to avoid saccades, it is not surprising to have a relatively large number of trials containing saccades or blinks shortly after stimulus onset.

Vergence was computed as the difference between the positions of the two eyes (Left eye position – Right eye position) and the mean vergence position during the baseline was subtracted from the profile for each trial. Segments of an epoch corresponding to saccades and blinks were eliminated and replaced via interpolation using 3^rd^ order splines. The resulting profiles were used to calculate vergence amplitude at the time of response (averaged over a time window of 20 ms) for each trial and participant. The vergence profiles were then averaged for each condition and participant by applying a Woody algorithm [32] and peak vergence amplitude was obtained for each profile. Vergence velocity profiles were obtained via numerical derivation and low-pass filtered using a 15 Hz cutoff. The onset of the vergence response was defined as the first moment in time when vergence velocity exceeded 10% of peak velocity for that trial. Finally, vergence latency (i.e., time from mask/step-stimulus onset to vergence-response onset) and mean vergence velocity were computed for each condition and participant. All statistical comparisons for the vergence data involved paired two-tailed *t* tests.

## Results

### Behavioral Data

**Figure 2:**
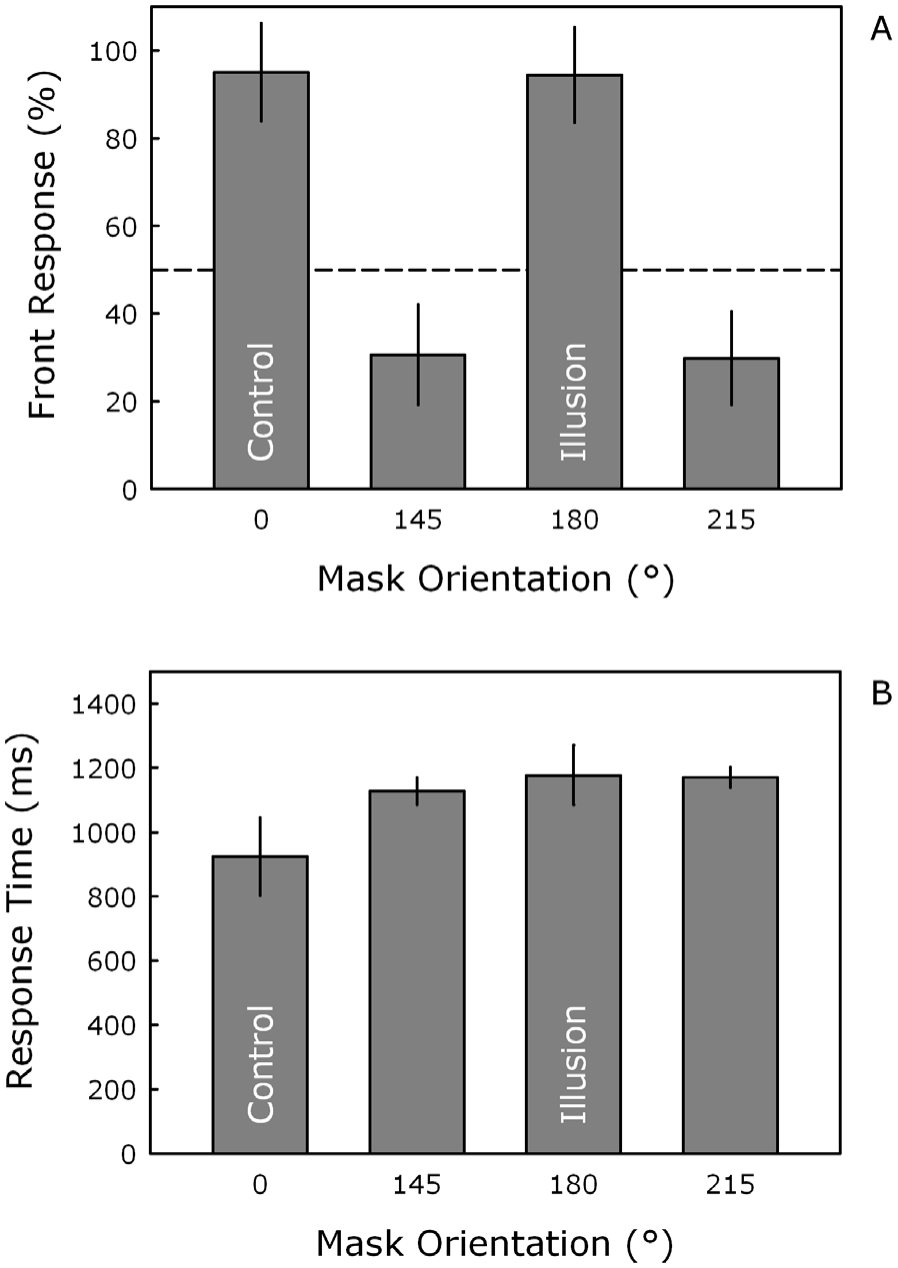
Behavioral data. Mean percentage of front responses (A) and mean response time for front responses (B) as a function of mask orientation (0°, 145°, 180°, 215°). The 0° and 180° mask orientations correspond to the control and illusion conditions, respectively. The dashed line in (A) refers to 50% and error bars correspond to ±1 standard error of the mean [37].

Figure 2A presents the mean proportion of front responses (in percent) for the different mask orientations. Participants almost always perceived the nose of the mask as pointing towards them (i.e., a convex face) in the control (0°) and illusion (180°) conditions, but not in the two filler conditions (145° and 215°). The ANOVA yielded a significant main effect of mask orientation, *F*(3, 24) = 14.86, η_p_^2^ = .65, *p*<.01, and contrasts confirmed that the control condition (95.1%) significantly differed from the two filler conditions (30.6% and 29.8%), both *t*s(8)>3.81, both *p*s<.01, but not from the illusion condition (94.4%), *t*(8) = 1.51, *p*>.16. These results clearly show that these participants experienced the hollow-mask illusion.

Mean response times for front responses are shown in Figure 2B. Response speed was not stressed and this was reflected in the relatively long response latencies (grand mean = 1099 ms). Although it appears that participants may have required somewhat less time in the control condition, there was no main effect of mask orientation when these values were submitted to the same analysis as above, *F*(3, 24) = 2.87, η_p_^2^ = .26, *p*>.11.

### Vergence Data

**Figure 3:**
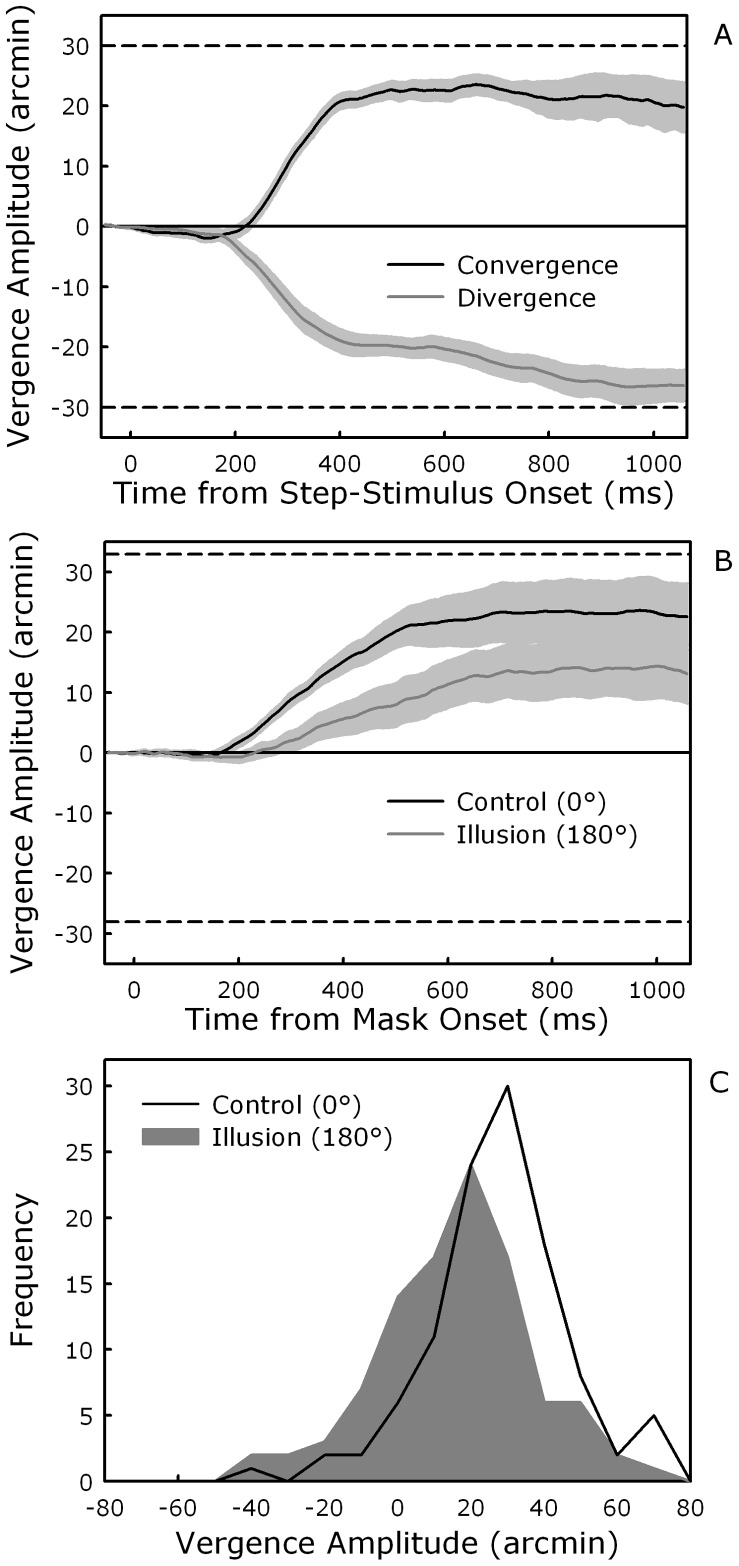
Vergence data. (A) Mean vergence amplitude for the convergent and divergent step stimuli as a function of time from step-stimulus onset (starting with a 50 ms baseline). The lines are encased in an envelope that corresponds to ±1 standard error of the mean. The upper and lower dashed lines mark the convergent and divergent vergence change that was expected based on the crossed and uncrossed disparities of the step-stimuli, respectively. (B) Mean vergence amplitude for front responses for the control (0°) and illusion (180°) mask orientations as a function of time from mask onset (starting with a 50 ms baseline). The lines are encased in an envelope that corresponds to ±1 standard error of the mean. The upper and lower dashed lines indicate the convergent and divergent vergence change that was expected based on the crossed and uncrossed disparities of the tip of the nose for the control and illusion conditions, respectively. (C) Histogram of the vergence amplitudes for front-response trials measured at the time of the button press for the control (0°) and illusion (180°) mask orientations.

Before turning to the mask stimuli, we briefly consider the convergent and divergent eye movements that were induced by the presentation of the vergence step stimuli (see Figure 3A). Participants approximated the expected values (dashed lines in the figure) and neither vergence latencies (grand mean = 212 ms), *t*(8) = 0.02, *p*>.97, nor absolute peak amplitudes (grand mean = 27 arcmin), *t*(8) = 1.15, *p*>.27, were different for convergence and divergence. Only mean velocities showed a tendency to be higher for convergence (0.94°/s) compared to divergence (0.65°/s), *t*(8) = 2.14, *p* = .06. Overall, these patterns correspond to normal vergence responses to convergent and divergent step stimuli [9], [33].

The mean vergence profiles (for front responses) in the control and illusion conditions are shown in Figure 3B. As expected, presentation of the convex (control) mask induced a convergent response that approached the virtual location of the tip of the nose (upper dashed line in the figure). In the illusion condition, the hollow mask induced a convergent response as well, which is inconsistent with the divergent vergence change that would have been expected based on the uncrossed disparities of the tip of the nose (lower dashed line in the figure). This overall pattern is, however, in line with participants’ illusory perception of a forward facing face (nose). That said, the vergence response in the illusion condition started on average later (251 ms vs. 166 ms after mask onset), *t*(8) = 3.71, *p*<.01, and was of smaller mean maximum amplitude (14 arcmin vs. 25 arcmin), *t*(8) = 2.99, *p*<.05, than in the control condition. Successive comparisons (paired *t* tests) of vergence amplitude at each time sample revealed that the two vergence responses started to significantly and systematically diverge from each other 216 ms after mask onset (for the use of a similar method, see, e.g., [34]). There were, however, no differences between conditions in the mean vergence velocity (grand mean = 0.75°/s), *t*(8) = 0.23, *p*>.81.

Although the mean vergence profile in the illusion condition never went in a divergent direction, its reduced amplitude compared to the control condition could reflect a mixture of convergent and divergent eye movements. To explore this issue, we focused on vergence amplitude at the time of response for each trial and participant. Consistent with the findings reported above, the mean amplitude was significantly higher in the control (21 arcmin) than in the illusion (10 arcmin) condition, *t*(8) = 3.62, *p*<.01. More importantly, the distributions of the vergence amplitudes were unimodal and similar in shape for both conditions, as shown in Figure 3C. This suggests that the difference in amplitude between the two conditions reflects a graded effect rather than the result of a mixture of distinct vergence responses (for a discussion of this logic, see [35]).

## Discussion

Previous research has suggested that people’s eyes converge at the perceived, rather than the actual, depth of the face when they experience the hollow-face illusion [11]. However, the nature of their measurements made it impossible to establish the exact timecourse of vergence eye movements and whether they were actually influenced by the presence of unambiguous binocular disparity information. Using a high-resolution eye tracker and a more controlled design, we were able to confirm that people’s eyes clearly converge, rather diverge, when they experience the hollow-face illusion. Moreover, the results of the vergence step-stimulus task allowed us to rule out that our participants were unable to properly respond to divergent disparity cues with an appropriate vergence adjustment. However, unlike what was reported by Hoffmann and Sebald [11], who only focused on rather coarse and late changes in vergence, the vergence response in the illusion condition was of smaller amplitude than in the control condition. The latter finding can be explained in at least one of two ways.

First, the integration of binocular disparity may have attenuated the magnitude of the illusion, such that the depth of the nose was perceived as being closer for normal (convex) than concave masks [5], [36]. According to this view, people converged at the depth they perceived, which suggests that both perception and action were subject to the illusion to the same extent. This interpretation is in line with that proposed by Hartung et al. [5] for pointing movements, although, in contrast to their study, we only assessed whether people experienced the illusion and not the actual depth at which they perceived the face to be.

A second, and alternative, account is that people perceived the tip of the nose as being at similar depths in both control and illusion conditions, but the vergence response itself was attenuated by the presence of conflicting depth information. This is potentially consistent with the notion that perception and vergence are based on separate processing streams (e.g., [15], [16]). However, we found that the onset of the vergence response in the illusion condition was delayed by 85 ms relative to the control condition. A delay which may have reflected the time it took the oculomotor system to override or inhibit a divergent response in the direction of the disparity-defined depth of the tip of the nose. Moreover, there was no indication that the eyes (initially) diverged when people experienced the illusion, which seems to speak against the second account, at least when pictorial cues are at the origin of the illusion of depth. This result is also noteworthy because it was previously suggested that vergence may have been one of the veridical cues that people relied upon in Króliczak et al.’s [6] study to produce accurate flicking movements to the face, even when they experienced the illusion. Thus, the dissociation they observed between conscious perception and object-oriented action probably stemmed from the reliance on a different cue, such as binocular disparity itself.

In sum, vergence responses in the illusion condition were both delayed and of smaller amplitude than in the control condition, which suggests that they were not immune to the influence of binocular disparity information. However, we found that the direction of vergence was clearly determined by perceived depth and not binocular disparity. This is overall line with Hoffmann and Sebald [11] and another recent study that focused on mean vergence position over prolonged viewing intervals [14], but also contrasts with a number of previous findings [15]–[17]. For example, Wismeijer et al. [15] employed the slant rivalry stimulus to create a conflict between monocular and binocular depth cues. Under such conditions, vergence responses always went in the direction of disparity-defined depths and were only somewhat attenuated when the perceived orientation conflicted with that indicated by the disparity cues (cf. [16]). Based on their findings, they concluded that depth cues, and not perceived depth, govern vergence. In light of the present findings, however, we are led to conclude that it actually depends on what you are looking at and, perhaps, on whether it is looking back at you.
